# Imaging of Glioblastoma Tumor-Associated Myeloid Cells Using Nanobodies Targeting Signal Regulatory Protein Alpha

**DOI:** 10.3389/fimmu.2021.777524

**Published:** 2021-11-30

**Authors:** Karen De Vlaminck, Ema Romão, Janik Puttemans, Ana Rita Pombo Antunes, Daliya Kancheva, Isabelle Scheyltjens, Jo A. Van Ginderachter, Serge Muyldermans, Nick Devoogdt, Kiavash Movahedi, Geert Raes

**Affiliations:** ^1^ Laboratory of Cellular and Molecular Immunology, Vrije Universiteit Brussel, Brussels, Belgium; ^2^ Myeloid Cell Immunology Lab, VIB Center for Inflammation Research, Brussels, Belgium; ^3^ Laboratory of Molecular and Cellular Therapy, Department of Biomedical Sciences, Vrije Universiteit Brussel, Brussels, Belgium; ^4^ In Vivo Cellular and Molecular Imaging Laboratory, Department of Medical Imaging, Vrije Universiteit Brussel, Brussels, Belgium

**Keywords:** signal regulatory protein alpha, glioblastoma, nanobodies (VHH), imaging, myeloid cell, SIRP alpha

## Abstract

Glioblastoma (GBM) is the most common malignant primary brain tumor. Glioblastomas contain a large non-cancerous stromal compartment including various populations of tumor-associated macrophages and other myeloid cells, of which the presence was documented to correlate with malignancy and reduced survival. *Via* single-cell RNA sequencing of human GBM samples, only very low expression of PD-1, PD-L1 or PD-L2 could be detected, whereas the tumor micro-environment featured a marked expression of signal regulatory protein alpha (SIRPα), an inhibitory receptor present on myeloid cells, as well as its widely distributed counter-receptor CD47. CITE-Seq revealed that both SIRPα RNA and protein are prominently expressed on various populations of myeloid cells in GBM tumors, including both microglia- and monocyte-derived tumor-associated macrophages (TAMs). Similar findings were obtained in the mouse orthotopic GL261 GBM model, indicating that SIRPα is a potential target on GBM TAMs in mouse and human. A set of nanobodies, single-domain antibody fragments derived from camelid heavy chain-only antibodies, was generated against recombinant SIRPα and characterized in terms of affinity for the recombinant antigen and binding specificity on cells. Three selected nanobodies binding to mouse SIRPα were radiolabeled with ^99m^Tc, injected in GL261 tumor-bearing mice and their biodistribution was evaluated using SPECT/CT imaging and radioactivity detection in dissected organs. Among these, Nb15 showed clear accumulation in peripheral organs such as spleen and liver, as well as a clear tumor uptake in comparison to a control non-targeting nanobody. A bivalent construct of Nb15 exhibited an increased accumulation in highly vascularized organs that express the target, such as spleen and liver, as compared to the monovalent format. However, penetration into the GL261 brain tumor fell back to levels detected with a non-targeting control nanobody. These results highlight the tumor penetration advantages of the small monovalent nanobody format and provide a qualitative proof-of-concept for using SIRPα-targeting nanobodies to noninvasively image myeloid cells in intracranial GBM tumors with high signal-to-noise ratios, even without blood-brain barrier permeabilization.

## Introduction

Glioblastoma (GBM) is the most prevalent and aggressive type of primary brain cancer. Standard treatment for GBM relies on surgical resection, radiotherapy and chemotherapy, but due to poor tissue accessibility, tumor invasiveness and rapid growth, patients relapse and the median survival is approximately two years following initial diagnosis. It is therefore imperative to find new diagnostic and therapeutic tools to tackle GBM (1). Over the last decade, immunotherapies such as the use of immune checkpoint inhibitors (ICIs), have revolutionized the field of cancer treatment (2). Cancer-immune cell interactions *via* so-called immune checkpoints, dampen anti-cancer immune responses and create an immuno-suppressive and pro-tumoral environment. Hence, the use of ICIs can promote anti-tumor immunity. Up to date, 7 ICIs that specifically focus on cytotoxic T cell activation have been clinically approved (3). Although these T-cell-centered ICIs have proven effective in so-called “hot” tumors such as melanoma and non-small cell lung carcinoma, which contain large proportions of cytotoxic T cells, only a minority of patients appears responsive to the treatment. Furthermore, they are of limited value in the treatment of non-T-cell inflamed “cold” tumors. Therefore, shifting the focus onto innate immune cells in order to boost anti-tumoral activity may provide complementary and synergistic potential for the treatment of tumors such as GBM, that, to date, only show very modest responses to the currently available ICIs (4, 5).

A potentially promising target is the SIRPα-CD47 axis (6). SIRPα is expressed by myeloid cells, including macrophages and dendritic cells, and binds to the ubiquitously expressed self-antigen CD47 (7). Their interaction serves as a “do not eat me” signal and avoids unwanted clearance of host cells. However, this mechanism is being exploited in the tumor microenvironment, as cancer cells overexpress CD47 to bypass macrophage-mediated phagocytic killing (8–10). Seminal pre-clinical mouse studies across many cancer types -including GBM- have shown that CD47-SIRPα interference significantly increases cancer cell engulfment (11–22). Consequently, several of such ICIs are currently being tested in clinical trials (23). Most studies are focusing on targeting CD47, using monoclonal antibodies. However, due to the ubiquitous expression of CD47, off-target adverse effects may arise. Secondly, as antibodies have a large molecular weight, their penetration capacity into brain tumors may be limited, for example in lowly vascularized hypoxic tumor regions or due to the presence of the blood-brain barrier (BBB). Therefore, specific targeting of SIRPα rather than CD47, and the use of smaller antigen-specific entities, may prove valuable in the context of GBM treatment.

Nanobodies are camelid-derived single-domain antibody fragments, which have emerged as promising tools for tumor targeting in both diagnostic and therapeutic settings (24–28). They are easily generated and retain high antigen specificity, but are smaller than monoclonal antibodies (29). Furthermore, preclinical studies have shown that nanobodies have superior tumor- and brain-penetrating capacity in comparison to monoclonal antibodies (30, 31).

In this study, we first confirm at single-cell resolution that SIRPα is a widely expressed target within the human and mouse GBM tumor microenvironment, with a high expression observed in tumor macrophages and certain dendritic cell (DC) subsets. Next, we generated SIRPα-specific nanobodies that bind the SIRPα^+^ tumor myeloid populations and revealed that the monovalent nanobody format can efficiently target mouse GBM tumors *in vivo*.

## Materials and Methods

### Expression, Purification and Quality Analysis of SIRPα Antigen

The gene encoding the ligand binding domain of mouse SIRPα (mSIRPα) (UniPROT ID P97797) was ordered at the company GenScript (Piscataway, USA) and cloned into the pHEN6 vector. *E. coli* bacteria (WK6 strain) transformed with the pHEN6-mSIRPα construct were inoculated in 5 mL of Luria-Bertani (LB) media supplemented with 100 µg mL^-1^ ampicillin (Sigma-Aldrich, St. Louis, USA), and were cultured overnight at 37°C, while shaking. On the next day, 1 mL of the bacterial suspension was transferred into 330 mL of fresh Terrific-broth (TB) media supplemented with 0.1% (w/v) glucose (Duchefa Biochemie, Haarlem, The Netherlands) and 100 µg mL^-1^ of ampicillin and cultured while shaking at 220 rpm and at 37°C for about 3 h until the OD_600nm_ reached 0.8. Isopropyl-β-D-thiolgalactopyranoside (IPTG) (Duchefa Biochemie, Haarlem, The Netherlands) was then added to the culture to a final concentration of 1 mM to induce the expression of the recombinant protein. The culture was incubated overnight at 28°C while shaking. The cells were harvested by centrifugation and the translocated recombinant periplasmic antigens were obtained *via* osmotic shock. Then periplasmic extracts underwent immobilized metal affinity chromatography (IMAC) using HisPur Ni-NTA resin (Thermo Fischer Scientific, Waltham, USA) as a capturing medium in a PD-10 column (GE-Healthcare, Chicago, USA) fitted with a filter. After washing with 20 column volumes of phosphate buffered saline (PBS), the HisPur bound SIRPα antigens were eluted in five 1 mL fractions of 500 mM imidazole (Merck, Darmstadt, Germany) in PBS (pH 7.5). The eluate was further purified by size exclusion chromatography (SEC) using a Superdex S75 16/600 column on a ÄKTA Express System (GE-Healthcare, Chicago, USA) using PBS as a mobile phase. The concentration of the SIRPα antigen was determined by spectroscopy at 280 nm with Nanodrop using the theoretically calculated extinction coefficient (32). The purity of the SIRPα antigens was further examined using sodium dodecyl sulphate polyacrylamide gel electrophoresis (SDS-PAGE).

### Generation and ELISA Screening of Nanobodies

Nanobodies were generated as previously described (33). An alpaca (*Vicugna pacos*) and two dromedaries (*Camelus dromedarius*) were immunized according to a six-week alternating schedule of weekly injections with 100 µg of recombinant antigen in Gerbu LQ 3000 adjuvant (GerbuBiotechnik GmbH, Heidelberg, Germany). Four days after the last immunization, 50 mL of anti-coagulated blood was collected from which plasma and peripheral blood lymphocytes were separated with Leucosep (Greiner Bio-One, Kremsünster, Austria) by density centrifugation. Total RNA was purified for the generation of cDNA by reverse transcription with oligo-dT primers. The generated cDNA was used as template in a two-step nested PCR that amplified the genes coding for the variable domains of the heavy-chain-only antibodies. The amplified pool of nanobody DNA fragments were ligated into a pMECS phagemid vector which were then transformed into *E. coli* TG1 electrocompetent cells. Using M13K07 helper phages, the nanobody libraries were expressed on phages. Enrichment for specific nanobody-phages was performed by 3 to 4 consecutive rounds of *in vitro* selection on recombinant antigen-coated wells of Nunc Maxisorp flat bottom microtiter plates (Thermo Fisher scientific). Clones were randomly selected from all rounds of panning of the different libraries and screened for binding on recombinant antigens using enzyme-linked immunoassays (ELISA).

Selected clones were sequenced and recloned into pHEN6 and pHEN25 plasmids for expression. The pMECS and pHEN6 vectors (to encode a C-terminal hexa-histidine (His6) tag respectively with or without an additional hemagglutinin (HA) tag) were used for expression of the monomeric version of the selected nanobodies, while the pHEN25 vector was used to obtain dimeric nanobodies. The pHEN25 expression vector is derived from the pHEN6 expression vector, where the nanobody amino-terminal glutamine is mutated to glutamic acid, followed by a fourteen amino acid long linker and a cysteine after the His6 tag, thereby allowing dimerization (24, 34).

For production, the pMECS-, pHEN6- and pHEN25-nanobody plasmids were transformed into a non-suppressor *E. coli* WK6 strain. The nanobodies were obtained *via* periplasmic extraction and subsequent purification by IMAC and SEC, as previously described. The concentration of the nanobodies was determined by spectroscopy at 280 nm with Nanodrop™ using the theoretically calculated extinction coefficient based on their amino acid sequence (32). The purity of all nanobodies was further examined using SDS-PAGE and western blot.

### Flow Cytometry Nanobody Screening

Purified nanobodies were screened for their ability to bind the cognate natively expressed antigen on the cell surface of the RAW 264.7 cell line (American Type Culture Collection, Manassas, USA). One µg of each nanobody was incubated with 2 x 10^5^ cells in DMEM (Gibco, Waltham, USA), supplemented with 10% FBS (Gibco), 300 mg mL^-1^ L-glutamine (Gibco), 100 U mL^-1^ penicillin and 100 mg mL^-1^ streptomycin (Gibco), at 4°C for 1 h. After incubation, the cells were washed three times with ice-cold PBS (pH 7.4) and then incubated with 1 µg of rat anti-mouse CD16/CD32 (clone 2.4G2, in house production) and 150 ng AF488 anti-mouse HA antibody (see **Supplementary Table 1**) prepared in ice-cold PBS (pH 7.4) and incubated for 30 min at 4°C in the dark. Finally, the cells were washed three more times, resuspended in 200 µL of ice-cold PBS (pH 7.4) and flow cytometry was performed on a FACS Canto II flow cytometer (BD Biosciences, San Jose, USA). As a negative control a nanobody against *Helicobacter pylori*’s outer-membrane adhesin BabA, i.e. Nb19, was used (35). APC anti-mouse CD172a (SIRPα) (clone P84, catalogue no. 144014, BioLegend, San Diego, USA) and APC rat IgG1 kappa isotype (clone RTK2071, catalogue no. 400412, BioLegend, San Diego, USA) antibodies were used as positive and negative controls for SIRPα expression detection on the RAW 264.7 cells. Data were analyzed using FlowJo 9.3.2 software (BD Biosciences). Based on forward and side scatter, cell debris and doublets were excluded, and the relative mean fluorescence intensity (ΔMFI) of AF488 was evaluated for each of the nanobodies compared to the anti-HA IgG.

### Kinetic Antigen Binding Profiles

The kinetic affinity parameters of the SIRPα nanobodies were determined by surface plasmon resonance (SPR) using a Biacore-T200 device (GE-Healthcare, Chicago, USA). The recombinant antigen was immobilized *via* amino coupling chemistry on a CM5 chip Series S (GE-Healthcare, Chicago, USA) at a concentration of 10 µg mL^-1^. The SPR measurements were performed at 25°C with HBS (20 mM HEPES pH 7.4, 150 mM NaCl, 0.005% Tween-20, 3.4 mM EDTA) as running buffer. The nanobodies were injected sequentially in 2-fold serial dilutions, from 500 to 1.95 nM. The rate kinetic constants were determined by a mathematical fitting of a 1:1 binding model using the Biacore Evaluation software, and the k_off_/k_on_ ratio was used to determine the equilibrium dissociation constant (K_D_).

### *In Vitro* Radioligand Binding Assay

^99m^Tc-labeled nanobodies were assessed for their ability to bind the cognate natively expressed antigen on the cell surface of the RAW 264.7 cell line (American Type Culture Collection, Manassas, USA). 5 x 10^4^ RAW 264.7 cells/well were allowed to adhere overnight in DMEM (Gibco, Waltham, USA), supplemented with 10% FBS (Gibco), 300 mg mL^-1^ L-glutamine (Gibco), 100 U mL^-1^ penicillin and 100 mg mL^-1^ streptomycin (Gibco). Cells were washed three times with ice-cold PBS (pH 7.4) and then incubated with 2 nM, 20 nM and 200 nM of ^99m^Tc-labeled Nb19 (5-51-514 µCi resp.; n=2), monovalent Nb15 (5-55-554 µCi resp.; n=2) and bivalent Nb15 (5-49-492 µCi resp.; n=2) for 1 h at 4°C. Aspecific binding was assessed by adding a 100x molar excess of unlabeled Nb. After incubation, the cells were washed 3 times with ice-cold PBS to remove unbound Nb, and cells were detached using 1 M NaOH (Merck, Darmstadt, Germany) and counted for radioactivity using a Wizard2 γ-counter (PerkinElmer, Massachusetts, USA). Specific binding was calculated as [counts per minute]_Unblocked_ – [counts per minute]_Blocked_.

### Mouse Imaging and Biodistribution Studies

#### Stereotactic Intracerebral Tumor Cell Inoculation

The GL261 cell line (36) was cultured in DMEM F12 (Gibco, Waltham, USA) supplemented with 10% FBS, 300 mg mL^-1^ L-glutamine, 100 U mL^-1^ penicillin and 100 mg mL^-1^ streptomycin. For intracranial injection, cells were harvested *via* trypsinization, brought to a concentration of 1 x 10^5^ cells mL^-1^ and injected in 7- to 10-week-old female C57BL/6J mice (Janvier) as previously described (37). Briefly, mice were anesthetized and immobilized in a stereotactic frame. A midline incision was made on the skin to expose the scalp and with a microdrill an injection burr hole was made. Then, very slowly, 5 x 10^5^ cells were injected using a Hamilton syringe. Tumors were allowed to grow for 21 days.

#### Preparation of ^99m^Tc-Labeled Nanobodies

Nanobodies were labeled with [^99m^Tc(H_2_O)_3_(CO)_3_]^+^ at their His6-tag *via* tricarbonyl chemistry, as described previously (38). The ^99m^Tc-labeled nanobodies were purified from the unbound [^99m^Tc(H_2_O)_3_(CO)_3_]^+^ *via* NAP-5 SEC (Sephadex, GE-Healthcare, Chicago, USA), and filtered through a 0.22 µm filter (Millex, Millipore, Burlington, USA). The radiochemical purity of radiolabeled nanobodies was evaluated by instant thin layer chromatography-silica gel (iTLC-SG, Pall Corporation, Belgium).

#### *In Vivo* Biodistribution of Radiolabeled Nanobodies

Mice bearing intracranial GL261 tumors were intravenously injected with ^99m^Tc-nanobodies (1-5-1.8 mCi; n=3). As a negative control, an anti-idiotypic nanobody targeting multiple myeloma, namely Nb R3B23, was used (25). Biodistribution analysis was performed as previously described (39). In brief, 1 h post-injection (p.i.), µSPECT/CT imaging was performed using a Vector^+^ MILabs system (MILabs, The Netherlands). SPECT-images were obtained using a rat SPECT-collimator (1.5-mm pinholes) in spiral mode, nine positions for whole-body imaging and three positions for brain imaging. Image analysis was performed using a Medical Image Data Examiner (AMIDE) software (40). After imaging, the mice were killed, and organs and tumors were isolated and weighed. The radioactivity in each sample was measured using a Wizard2 γ-counter (PerkinElmer, Massachusetts, USA). Tracer uptake was expressed as % injected activity per gram organ (%IA/g). Statistical analyses were performed using one-way ANOVA.

### Flow Cytometry Nanobody Binding on *Ex Vivo* Tumors

Tumor tissue was processed into single cell suspension as previously described (41). Thereto, at 21 days post tumor inoculation, mice were sacrificed and transcardially perfused with 20 mL of ice-cold PBS. Using a stereomicroscope, tumors were carefully dissected from the surrounding brain. They were cut into small pieces and incubated with enzyme mix (30 U mL^-1^ DNAse I (Roche), 10 U mL^-1^ collagenase type I (Worthington) and 400 U mL^−1^ collagenase type IV (Worthington), diluted in 1x HBSS (Gibco)), in a 1:3 ratio with RPMI (Gibco) for 20 min at 37°C. Subsequently, tumor tissue was crushed with a syringe plunger and heavily triturated using standard serological pipettes. The homogenized tissue was filtered twice over a 100 µm nylon filter and centrifuged (515 g, 5 min, 4°C). The pellet was resuspended in red blood cell lysis buffer (155 mM NH_4_CL, 12 mM NaHCO_3_ and 0.1 mM EDTA (Duchefa), dissolved in PBS). After 3 min, the lysis reaction was quenched by adding 9 mL of RPMI (Gibco), samples were centrifuged (450 g, 5 min, 4°C) and the pellet was resuspended in FACS buffer (2 mM EDTA, 2% heat-inactivated FCS (Gibco), dissolved in 1x HBSS). The cells were incubated for 15 min on ice with Zombie Aqua Fixable Live-Dead stain (BioLegend) at a 1:1000 concentration. Next, samples were washed, and incubated on ice for 1h with 5 µg of His6-tagged Nb15 or control Nb19. Samples were then washed again, blocked with rat anti-mouse CD16/CD32 (clone 2.4G2) for 15 min on ice and stained with fluorescently labeled antibodies for 30 min on ice. The antibodies that were used are listed in **Supplementary Table 1**. After a final wash step, flow cytometry was performed on a FACS Canto II flow cytometer and data was analyzed using FlowJo software.

### Screening of SIRPα and CD47 Expression in Published Mouse and Human GBM Datasets

The expression matrices of single-cell RNA sequencing (scRNA-seq) of newly diagnosed (ND) human GBM tumors (patients ND1-6), of CITE-seq of newly diagnosed and recurrent (R) human GBM tumors (patients R2, R5 and ND8), and of CITE-seq of mouse GL261 tumors, previously generated in our lab (40), have been downloaded. The single cell data has been analyzed as previously described (40). Batch correction has been applied for the human ND-GBM scRNA-seq and for the human GBM Citeseq using harmony v1.0 with theta of 1 (theta is a diversity clustering penalty parameter with a default value of 2, higher theta resulting in a more aggressive correction). Unsupervised clustering has been performed using the Leiden algorithm. For the human ND-GBM scRNA-seq, the first 30 harmony-corrected PCA embeddings and resolution 0.25 were used for the clustering, yielding 13 clusters, which were annotated as cancer cells, myeloid cells, T cells, oligodendrocytes, endothelial cells and fibroblasts. The human GBM CITE-seq dataset was clustered using the first 30 harmony-corrected PCA embeddings and resolution 1. Artefact and doublets clusters were filtered out. For the clustering of the mouse GL261 CITE-seq, the first 30 PCA embeddings and resolution 1 were applied. Doublet cells, co-expressing macrophage genes and markers of other cell types were manually removed.

To estimate the effect of sex on SIRPα expression in the myeloid compartment of the GBM tumors, we evaluated scRNAseq data from 6 female (ND1, ND6-8, R1-2) and 7 male (ND2-5, R3-5) GBM patients. The myeloid cells from these datasets were extracted (excluding mast cells), the expression was normalized per cell and the average SIRPα expression per patient was calculated.

## Results

### The Immune Checkpoint SIRPα Is Highly Expressed by Tumor-Associated Myeloid Cells in Both Human and Mouse GBM

**Figure 1:**
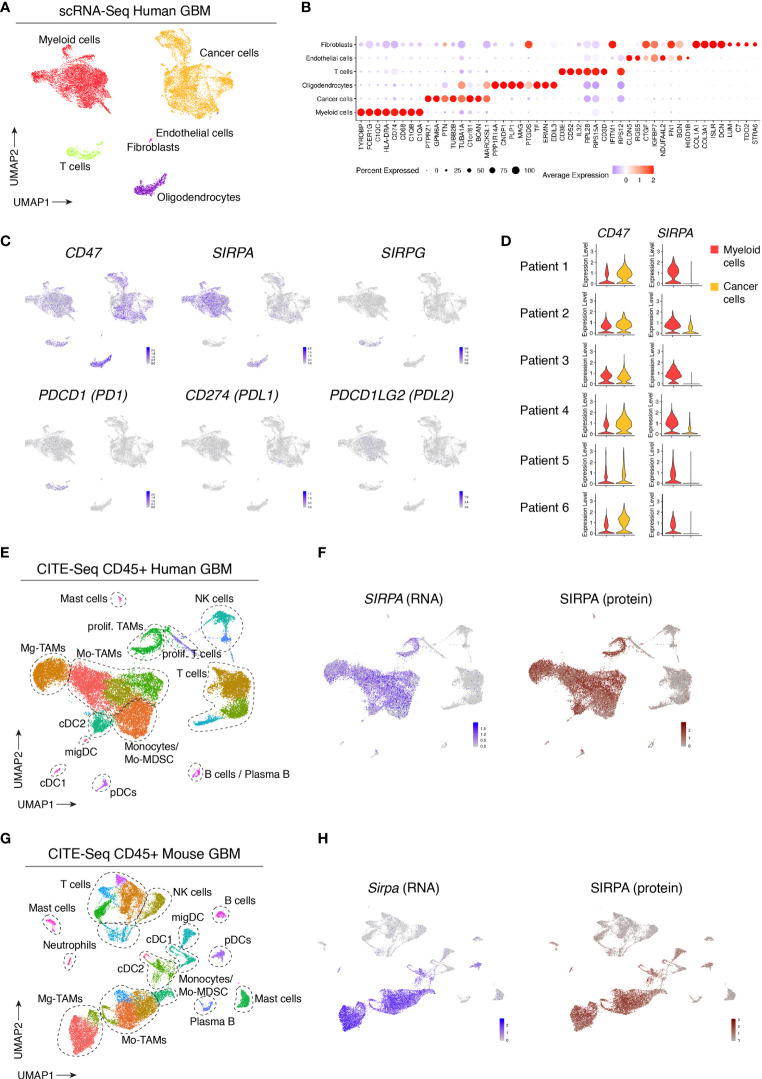
Single-cell profiling of human and mouse GBM tumors reveals consistent SIRPα gene and protein expression in tumor-associated myeloid cells. **(A)** UMAP plot of 20033 cells isolated from n = 6 GBM tumors, visualizing the identified cell populations. **(B)** Dot plot, corresponding to the UMAP plot shown in **(A)**, visualizing the expression of key signature genes of the indicated cell populations. The dot size relates to the percentage of cells expressing the gene, while the color relates to its scaled average expression. **(C)** UMAP plots showing expression of the indicated genes. The color code and range of normalized counts are shown at the lower right on each plot. **(D)** Violin plots showing the expression of *CD47* and *SIRPA* on myeloid cells (red) and cancer cells (yellow) for each individual patient. **(E)** Gene expression-based UMAP plot of 25113 CD45^+^ cells, isolated from n = 3 human GBM tumors and profiled with CITE-seq. **(F)** Feature plot showing *SIRPA* gene expression (blue) and SIRPA protein expression (brown) based on CITE-seq antibody staining (Antibody-Derived Tag or ADT signals), corresponding to the dataset shown in **(E)**. **(G)** Gene expression-based UMAP plot of 23926 CD45^+^ cells isolated from orthotopic mouse GL261 tumors (n = 2 groups) and profile with CITE-seq. **(H)** Feature plot showing *Sirpa* gene expression (blue) and SIRPA protein expression (brown) in GL261 tumors based on CITE-seq antibody staining, corresponding to the dataset shown in **(G)**.

To evaluate the expression and distribution of SIRPα within the glioblastoma (GBM) microenvironment, we reanalyzed our recently published single-cell RNA sequencing (scRNA-seq) and cellular indexing of transcriptomes and epitopes sequencing (CITE-seq) datasets of human and mouse GBM (41). While scRNA-seq provides information about the transcriptome of individual cells, CITE-seq additionally uses a panel of barcoded antibodies which allows for the simultaneous quantification of both mRNA and cell surface protein expression (42). Unbiased scRNA-seq analysis of six newly diagnosed human GBM tumors revealed various cancer and stromal cell populations (**Figure 1A**). Based on known marker genes, we identified cancer cells, immune cells, oligodendrocytes and small clusters of fibroblasts and endothelial cells (**Figures 1A, B**). *SIRPα* expression was limited to tumor oligodendrocytes and myeloid cells, while its ligand, *CD47*, was ubiquitously expressed within the tumor microenvironment (**Figure 1C**). Results were consistent across all patients (**Figure 1D**). The expression of *SIRPG*, which also binds to CD47, was restricted to T cells (**Figure 1C**). Concerning prototypical immune checkpoints, expression of *CD274* (*PDL1*) and *PDCD1LG2* (*PDL2*), which encode ligands for *PDCD1* (*PD1*), expressed in tumor-infiltrating T cells, was virtually absent in the profiled human GBM tumors (**Figure 1C**). To assess whether *SIRPα* was differentially expressed in females *versus* males, we compared its expression in myeloid cells from newly diagnosed and recurrent patients, which showed no significant difference between males and females in this cohort of 13 patients (**Supplementary Figure 1**). To obtain a more detailed overview of *SIRPα* gene and protein expression in immune cells, we analyzed the CD45^+^ compartment of 3 human GBM tumors *via* CITE-seq (41). Immune cell clusters were identified as previously described (41). Within the immune cell compartment, *SIRPα* was primarily expressed by dendritic cells (DCs), mainly the type 2 conventional (cDC2) subset, monocytes and tumor-associated macrophages (TAMs) (**Figures 1E, F**). As shown previously (41), TAMs in GBM tumors can be derived from microglia (Mg-TAMs) or monocytes (Mo-TAMs) (**Figure 1E**). Both subsets expressed *SIRPα* at comparable levels, and a close correlation between mRNA and protein expression was revealed (**Figure 1F**). To assess *SIRPα* expression in mouse GBM tumors, we reanalyzed the CITE-seq dataset from the CD45^+^ fraction of orthotopic GL261 tumors (41). This yielded comparable results as in human tumors, with robust SIRPα gene and protein expression observed in TAMs and DCs, primarily cDC2s (**Figures 1G, H**).

Together, these results identified SIRPα^+^ myeloid cells -in particular cDC2s, monocytes and TAMs- as a potentially relevant target population in human GBM, and verify that mouse GBM functions as a good model system in this context.

### mSIRPα Targeting Nanobodies Bind to Recombinant and Cell-Membrane mSIRPα *In Vitro*

**Figure 2:**
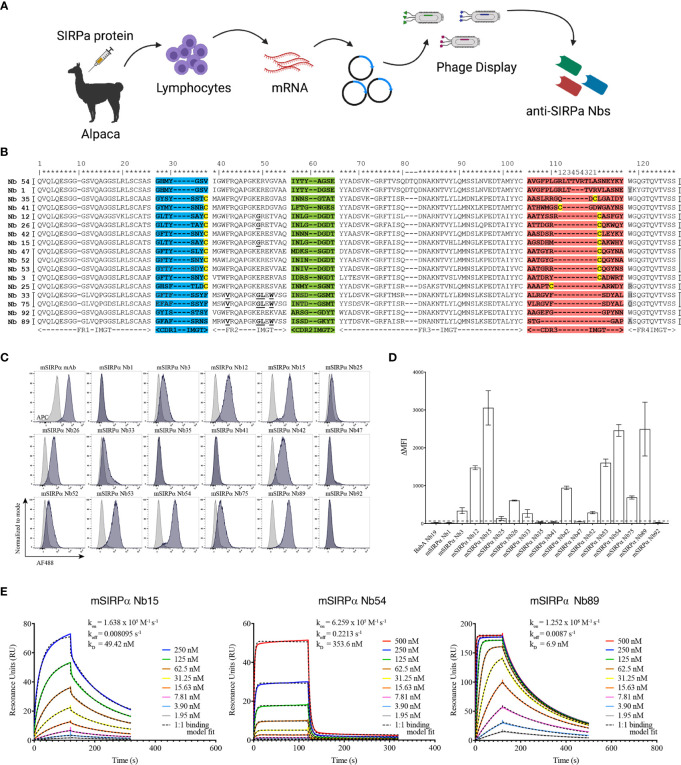
Single-domain antibodies targeting SIRPα can be obtained following immunization with recombinant SIRPα protein. **(A)** Schematic representation of nanobody generation procedure. A camelid is immunized with recombinant SIRPα protein, the mRNA of peripheral blood lymphocytes is converted to cDNA and the region encoding the antigen binding domain of the camelid heavy chain-only antibodies is amplified by PCR and cloned in a phage display vector. Antigen-specific nanobodies are retrieved after phage display and panning on plastic coated SIRPα. **(B)** Amino acid sequence of mSIRPα nanobodies, with numbering according to the International ImMunoGeneTics – IMGT – information system http://www.imgt.org (43). The CDR1, CDR2 and CDR3 regions are colored in blue, green and red, respectively. The cysteine residues used as subfamily-hallmarks are highlighted in yellow. The amino acids which differ from the typical VHH hallmark residues in framework-2 are in bold and underlined. The amino acid at position 118 (Trp) is highly conserved, however, in nanobodies this amino acid is sometimes substituted, usually with Arg and highlighted here in grey. Each nanobody family is based on the sequence identity of the CDR3 region and nanobodies belonging to the same family are grouped, indicated by the vertical black lines on either side of the sequence (14 families in total). Asterixes on top are used to indicate amino acid positions. **(C)** Representative histogram plots of mSIRPα nanobodies binding to RAW 264.7 macrophages. Overlay of binding signals of mSIRPα nanobodies (blue) versus a non-targeting nanobody BabA Nb19 (grey). Note: the first plot shows binding of monoclonal antibody targeting mSIRPα (positive control, blue) and mouse IgG (isotype control, grey) **(D)** Median fluorescence intensity (the difference between the signal of the nanobody and the signal of the mouse anti-HA IgG) of the mSIRPα nanobodies binding to RAW 264.7. The dashed black line is defined by the triple ΔMFI value of the non-targeting nanobody (BabA Nb19) and it represents the threshold above which a nanobody is considered to bind specifically to its target. **(E)** Kinetic rate constants determination by SPR: the sensorgrams of different concentrations (as indicated in the inserts) of mSIRPα nanobodies binding to the recombinant antigen. Kinetics were measured with two-fold serially diluted nanobodies and the fitting of the binding curves was using a 1:1 binding mathematical model.

Nanobodies were generated against the recombinant extracellular domain of mouse SIRPα (**Figure 2A**). ELISA screenings and sequencing of individual clones led to the identification of 17 individual nanobody clones binding to mouse SIRPα (mSIRPα), belonging to 14 clonally unrelated nanobody families, based on the sequence identity of the CDR3 (**Figure 2B**). Among these, 12 were able to bind to native murine SIRPα-expressing RAW 264.7 macrophages in flow cytometry (**Figures 2C, D**), with clones Nb15, Nb54 and Nb89 exhibiting the highest median fluorescence intensities (**Figure 2D**). Surface plasmon resonance (SPR) measurements of these 3 latter nanobody clones revealed binding affinities between 6,9 and 353,6 nM (**Figure 2E**). Epitope binning revealed that these 3 nanobodies did not compete with each other for antigen binding, except for a partial inhibition of each other’s binding by Nb54 and Nb89, suggesting they mainly recognize non-overlapping epitopes on the antigen (**Supplementary Figure 2**).

### Biodistribution Studies in the Mouse GL261 GBM Model Reveal Nb15 as a Prime Candidate for *In Vivo* Tumor Targeting of mSIRPα

To analyze their *in vivo* targeting potential, the 3 selected mSIRPα nanobodies, and a non-targeting control nanobody were labeled with ^99m^Tc. All the ^99m^Tc-labeled nanobodies had a radiochemical purity larger than 95%. Biodistribution and tumor targeting of ^99m^Tc-nanobodies were assessed in mice bearing GL261 brain tumors, *via* micro-SPECT/CT at 1 hour post injection. Kidneys showed overall the highest signal irrespective of the nanobody, reflecting the fast filtration of unbound nanobody from the bloodstream, as is often observed for other nanobodies (44).

**Figure 3:**
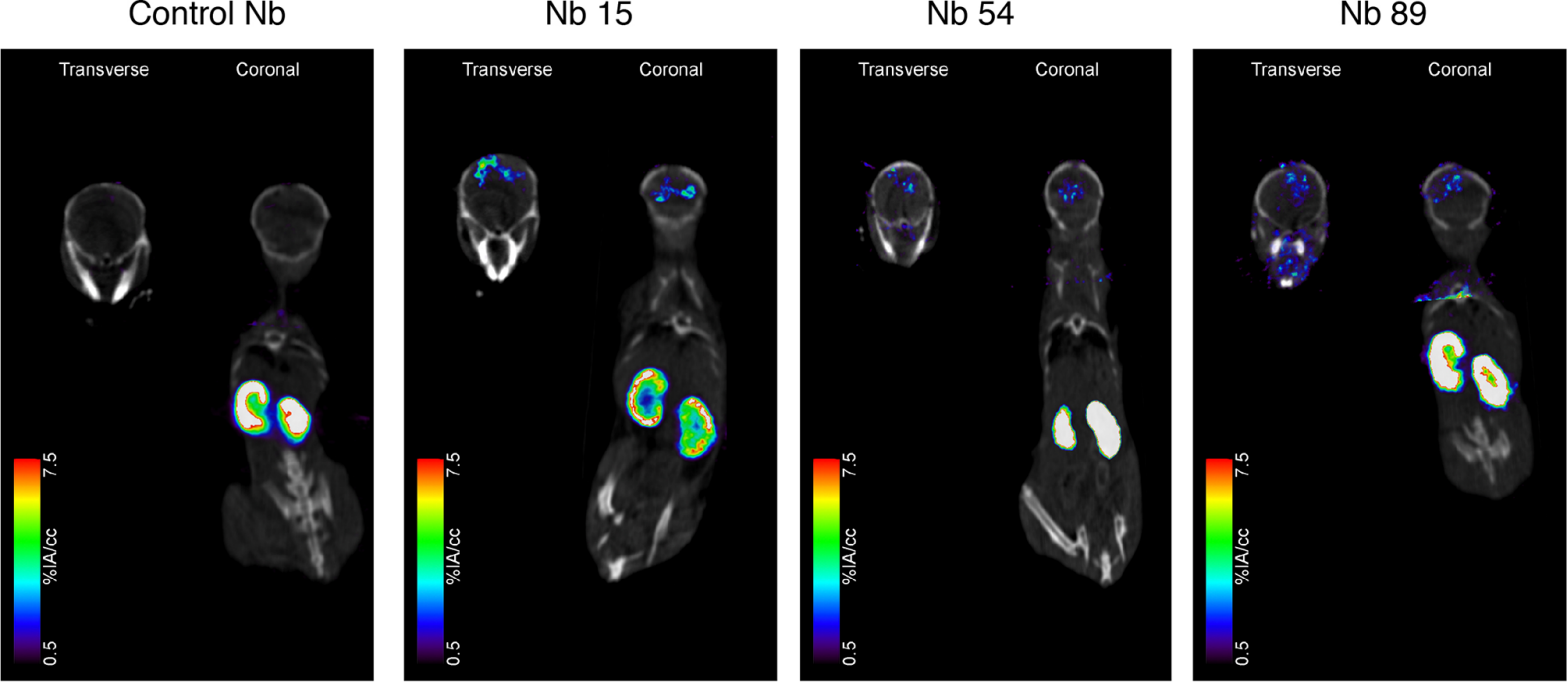
Anti-SIRPα Nb15 targets mouse GL261 GBM tumors *in vivo*. Fused pinhole SPECT/micro-CT images of GL261 tumor-bearing mice injected with ^99m^Tc-labeled “anti-SIRPα Nb clones 15, 54 and 89 or a non-targeting control Nb R3B23. Mice were imaged 1 hour post injection. Transverse and coronal views are shown, with slices chosen to pass through the brain tumor, without taking other organs into account. Slices that explicitly go through other organs are shown in **Supplementary Figure 3**. Results are representative of n = 3 mice for each group.

Among the tested anti-mSIRPα nanobodies, Nb15 exhibited the most profound tumor targeting potential as compared to the control nanobody (**Figure 3** and **Table 1**). Additionally, Nb15 showed high uptake in spleen, liver, lungs, thymus, lymph nodes and bone, while lower background signals were noted for other tissues (**Table 1** and **Supplementary Figure 3**). Signals detected in peripheral organs such as lungs and spleen were significantly lower for Nb89 and Nb54 as compared to Nb15. These data point to an inherently better *in vivo* targeting and imaging potential of Nb15 as compared to Nb89 and Nb54. Hence, even without forced BBB permeabilization, Nb15 allowed us to clearly image mSIRPα in intracranial GBM tumors, exhibiting high signal-to-noise ratios (**Figure 3** and **Table 1**).

**Table 1:** Uptake values of ^99m^Tc-labeled control Nb R3B23 or anti-SIRPα nanobodies in GL261 tumor-bearing mice based on dissection at 1 h 45 min post injection.

	Control Nb	Nb15	Nb54	Nb89
**Blood**	1.8055 ± 0.0315	1.3985 ± 0.3665	1.141 ± 0.3160	1.2375 ± 0.0335
**Thymus**	0.8435 ± 0.0225	1.3950 ± 0.5120	0.5000 ± 0.1000	0.6595 ± 0.0135
**Heart**	0.6850 ± 0.0320	1.2005 ± 0.2235	0.4745 ± 0.1315	0.6920 ± 0.0920
**Lungs**	0.8730 ± 0.7350	3.0315 ± 0.7025	1.0245 ± 0.2215	1.712 ± 0.0810
**Liver**	0.9150 ± 0.0090	3.0450 ± 0.5120	3.5060 ± 0.4880	4.3990 ± 0.0090
**Spleen**	0.7170 ± 0.0750	8.9050 ± 2.6080	1.7785 ± 0.2935	2.1655 ± 0.3385
**Pancreas**	0.4815 ± 0.0465	0.6945 ± 0.2005	0.3305 ± 0.0525	0.5070 ± 0.047
**Left kidney**	295.3835 ± 3.3245	269.206 ± 47.384	303.6065 ± 23.9875	265.543 ± 7.931
**Right kidney**	306.5695 ± 7.1735	283.6185 ± 28.0615	278.9795 ± 20.2255	283.552 ± 10.1700
**Muscle**	0.7640 ± 0.3150	0.3360 ± 0.0740	0.1720 ± 0.0220	0.3725 ± 0.0705
**Bone**	0.5565 ± 0.1745	1.8685 ± 0.0055	0.3855 ± 0.0345	0.8250 ± 0.0100
**Lymph nodes**	0.8680 ± 0.1360	1.5695 ± 0.4175	0.5885 ± 0.2035	0.9560 ± 0.1310
**Brain**	0.1355 ± 0.0265	0.4080 ± 0.3590	0.1165 ± 0.0535	0.1100 ± 0.0160
**GL261 tumor**	0.8080 ± 0.0260	1.7195 ± 0.2125	0.5010 ± 0.2290	0.6725 ± 0.0195

### Nb15 Targets Monocytes and Tumor-Associated Macrophages From Mouse GBM Tumors

**Figure 4:**
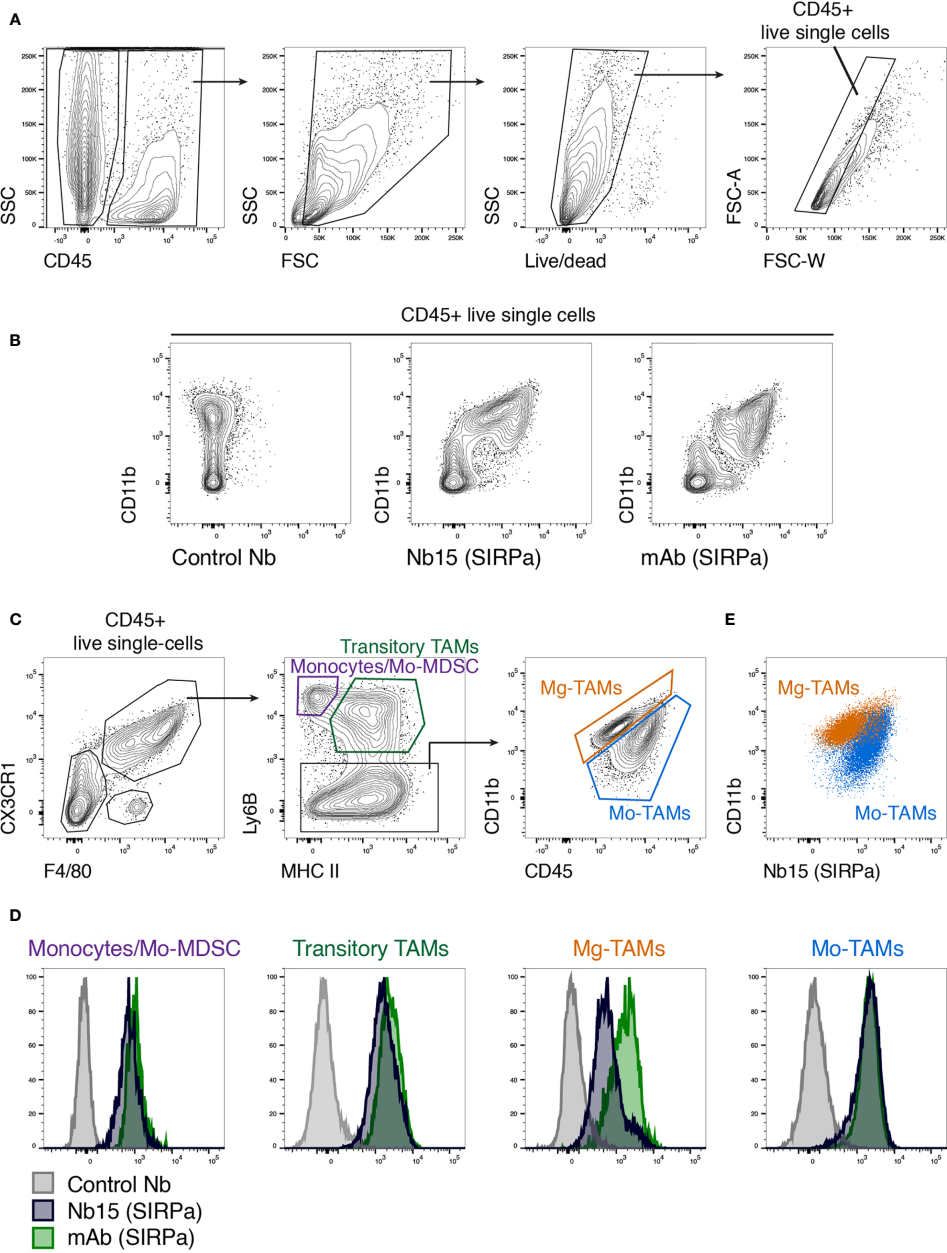
*Ex vivo* staining confirms the specificity of anti-SIRPα Nb15 for tumor-associated monocytes and macrophages. **(A)** Single-cell suspensions were made from orthotopic GL261 tumors, followed by flow cytometric analysis. CD45^+^ live single cells were gated as indicated. **(B)** Flow cytometry plots showing staining with CD11b in combination with either a non-targeting control nanobody, an anti-mSIRPα Nb15 or a commercially available monoclonal anti-mSIRPα antibody. Cells were pre-gated on CD45^+^ live single cells. **(C)** Tumor-infiltrating monocyte and macrophage populations were gated based on their expression of CX3CR1, F4/80, Ly6B, MHC-II, CD11b and CD45, as indicated. Monocyte-derived or Mo-TAMs and microglia-derived or Mg-TAMs were distinguished based on their differential expression of CD11b versus CD45. **(D)** Histogram plots showing staining with a commercially available anti-mSIRPα mAb (green), anti-mSIRPα Nb15 (blue) or a non-targeting control Nb19 (grey) for the indicated populations [gated as shown in **(C)**]. **(E)** CD11b and anti-mSIRPα Nb15 staining in Mo-TAMs (blue) and Mg-TAMs (orange) were overlaid to reveal their differential expression. Results are representative of n = 4 mice.

After identifying Nb15 as a suitable probe for *in vivo* imaging of mSIRPα in GBM tumors, we wanted to evaluate its binding capacity on the various populations of mSIRPα expressing tumor-infiltrating myeloid cells. Hereto, GL261 tumors were microdissected and processed into single-cell suspensions, whereupon Nb15 binding was assessed *via* flow cytometry. First, CD45^+^ cells were selected followed by the exclusion of debris, dead cells and doublets (**Figure 4A**). Within the CD45^+^ live single cells, the myeloid cells (CD11b^+^) exhibited clear binding of Nb15, when compared to a control nanobody (**Figure 4B**). Within CX3CR1^+^ F4/80^+^ cells, we subgated on monocytes (MHCII^-^ Ly6B^+^), transitory TAMs (MHCII^+^ Ly6B^+^) and mature TAMs (Ly6B^-^) (**Figure 4C**), using a previously described gating paradigm (41). Nb15 showed efficient binding to these different populations, in comparison to the control nanobody (**Figures 4D, E**). These results confirmed that Nb15 efficiently bound to SIRPα^+^ monocytes and TAMs in GL261 tumors.

### Monovalent Nb15 Is the Preferred Format for *In Vivo* Tumor Imaging in Mice

**Figure 5:**
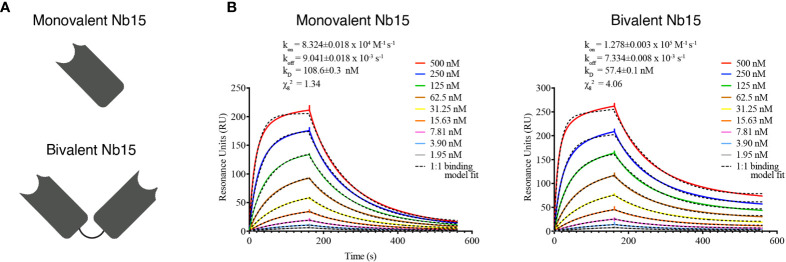
Bivalent anti-SIRPα Nb15 binds SIRPα. **(A)** Schematic representation of mono- and bivalent anti-SIRPα Nb15. **(B)** Kinetic rate constants determination by SPR: the sensorgrams of different concentrations (2x serial dilution) of mono- and bivalent anti-SIRPα Nb15 binding to the recombinant antigen. Fitting of the binding curves was obtained by using a 1:1 mathematical model, for the mono- and bivalent constructs. Kinetic parameters are included as mean±SD.

**Figure 6:**
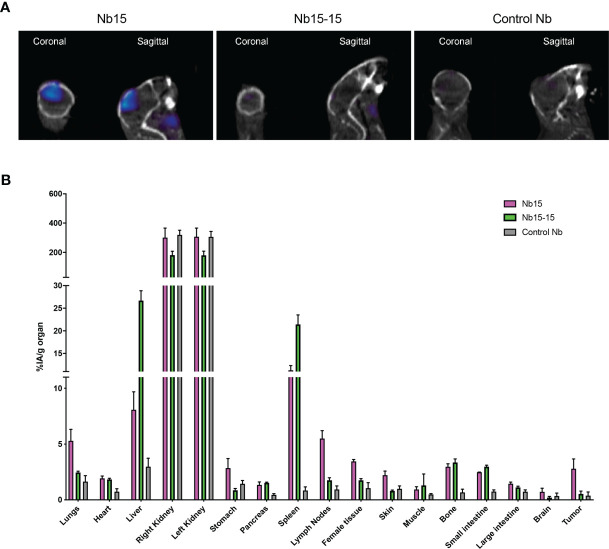
Bivalent anti-SIRPα Nb15 exhibits impaired tumor targeting. **(A)** Fused pinhole SPECT/micro-CT images of GL261 tumor-bearing mice, inoculated with GL621 at the same time and randomized before injection of ^99m^Tc-labeled monovalent or bivalent anti-SIRPα Nb15 or a non-targeting control Nb R3B23. Mice were imaged 1 hour post tracer injection. Coronal and sagittal views are shown. Images are representative of n = 3 mice for each group. Similar results were obtained for the different mice in each group. **(B)** *Ex vivo* radioactivity values measured in the indicated dissected organs at 1 hour 45 min post injection with ^99m^Tc-labeled monovalent (magenta) or bivalent (green) anti-SIRPα Nb15 or non-targeting control Nb R3B23. Values are expressed as injected activity per gram (%IA/g). Results are represented as mean ± SEM of n = 3 mice for each group.

The above-mentioned *in vitro, in vivo* and *ex vivo* data support that Nb15 could serve as a potent GBM tumor-targeting tool. Previous reports have shown that the binding capacity of nanobody constructs can significantly improve upon self-coupling, due to increased avidity (45). To examine this for Nb15, we created bivalent constructs (**Figure 5A**). *In vitro* characterization by surface plasmon resonance and radioligand binding assay, confirmed a robust binding capacity of both the monovalent and bivalent construct to the antigen (**Figure 5B** and **Supplementary Figure 4**). *In vivo* distribution, however, showed that the tumor targeting capacity of Nb15, was completely abolished by the creation of a bivalent construct (**Figure 6A**). Radioactivity measurements of brain, tumor and peripheral tissues, showed that tissue targeting of the bivalent construct was higher as compared to the monovalent construct in SIRPα^+^ peripheral organs such as the liver and spleen. In the tumor, on the other hand, radioactivity dropped to similar background levels as with the control nanobody (**Figure 6B**). This reveals that small targeting moieties more efficiently penetrate GBM tumors.

## Discussion

### Nanobodies as Efficient Tools for *In Vivo* Imaging of mSIRPα in GBM Tumors

In the present manuscript, we describe the generation and characterization of nanobodies against SIRPα, as targeting agents for SIRPα-positive glioblastoma (GBM)-infiltrating myeloid cells. Nanobodies isolated from immune libraries obtained after immunization with the recombinant ectodomain of SIRPα, were subjected to a cell binding screening using flow cytometry to determine their ability to bind the native form of the antigen. This revealed that 12 of the nanobodies could recognize murine macrophages expressing SIRPα. Among the 3 nanobodies exhibiting the highest median fluorescence intensities for binding to mouse SIRPα in flow cytometry, Nb15 was found to also target orthotopically implanted GL261 tumors *in vivo*, as shown *via* SPECT-CT imaging and biodistribution analysis of ^99m^Tc-labeled nanobodies.

Most brain diseases and tumors structurally disrupt the BBB, consequently making it more permeable and easier to cross. GBMs, and in particular also in the GL261 murine GBM model used in this study, are known to display increased BBB disruption as they progress (46, 47). Importantly, Nb15 could target the GBM tumors even in the absence of additional BBB permeabilization. The nanobody format in this situation could be an advantage overcoming some of the limitations of the conventional antibodies, such as their slow diffusion through tissues and large size (150 kDa), even with a disrupted BBB that occurs in this type of disease and disease model. Nanobodies with their small size (15 kDa) and favorable pharmacokinetic properties could hypothetically have an easier path on their way to reach brain targets (48, 49). Moreover, there have already been reports of nanobodies passing the BBB, such as for example nanobodies targeting the prion protein (50), targeting Aβ fibrils associated with Alzheimer disease (51) or targeting tumor antigens associated with brain tumors (30, 31).

For Nb54 and Nb89, the accumulation in the GBM tumor did not significantly exceed that of the control nanobody. This difference in uptake was not correlated with the affinity for the target, since the affinity of Nb89 for mSIRPα as detected *via* SPR was even higher as compared to that of Nb15. It has been suggested that nanobodies with a basic pI could cross the BBB (52). However, this did not seem to be a factor here either, since Nb89 had an EXPASY calculated theoretical pI of higher than 9, as compared to below 7 for Nb15. The observation that Nb15 also showed high uptake in spleen, liver, lymph nodes, thymus, bone (marrow) and lungs, which are known macrophage resident “hot spots”, indicates that the differences in GBM tumor targeting potential between these nanobodies was most likely related to an inherently better *in vivo* targeting and imaging potential of Nb15 as compared to Nb89 and Nb54. In particular for Nb54, its fast off-rate may contribute to a poor *in vivo* targeting. Possibly, the targeted epitope could be important for effective *in vivo* targeting of Nb15 to SIRPα on myeloid cells.

### Possible Implications for Diagnostic Applications

Whole body preclinical SPECT/CT imaging using radiolabeled nanobodies targeting SIRPα as performed in the current study provides a proof of concept for *in vivo* targeting of SIRPα on GBM tumor-infiltrating myeloid cells and confirms the added value and favorable pharmacokinetics of monovalent nanobodies. Monovalent nanobodies offer a rapid targeting to antigen-positive organs, followed by fast clearance of non-targeting probes *via* the kidneys. This yields a high signal-to-noise contrast and limited off-target radiotoxicity, allowing high contrast imaging within 1 h post injection. Given that TAMs and the markers they express have been documented to correlate with malignancy and reduced survival in GBM patients (53), nanobody-based detection of SIRPα in GBM may entail prognostic value. Thereby, a high accumulation of radiolabeled nanobodies targeting SIRPα may correlate with the presence of a high amount of immune suppressive TAMs. Alternatively, a higher signal may correlate with higher expression of SIRPα per cell, reflecting a more immune suppressive environment, but potentially also rendering the TAMs more responsive to SIRPα-targeted therapies. As such, nuclear imaging of SIRPα may have value for disease monitoring or therapy guidance in GBM.

Besides whole body imaging, nanobodies also offer diagnostic possibilities for image-guided surgery (26). In our recently documented efforts to unravel the GBM immune landscape (41), multiplex immunohistochemistry revealed that (SIRPα expressing) TAMs are found throughout human GBM tumor tissue. Thus, an interesting perspective is that fluorescently labeled nanobodies targeting SIRPα could be evaluated for delineating tumors during surgery (26).

As a remark, while the current study provides a first qualitative indication that Nb15 can be used to target myeloid cells in a glioma model, proof that the method can also be used in a quantitative manner to track accumulation of SIPRα-expressing myeloid cells or to monitor upregulation of SIRPα expression has not been provided. This will need to be addressed in follow-up studies. Thereby, myeloid cell depletion strategies could help to evaluate whether the technique could be employed to quantify the abundance of myeloid cells in the glioma microenvironment. Corresponding IHC/IF of tumor area or *ex vivo* flow cytometry analysis of SIRPα expression could also be used to assess how well the radioactive signals from the tracer and the expression of the marker match.

### Possible Implications for Therapeutic Applications

A significant body of evidence supports the targeting of the CD47-SIRPα immune checkpoint as a promising strategy against several hematological and solid tumors, especially when used in combination with other inhibitors targeting T-cell immune checkpoints, such as PD-L1-PD-1 (54–56). In GBM, preclinical data indicate that blocking the CD47-SIRPα axis can induce antitumor effects (13, 14, 57), although a combination with chemotherapy may be required to activate ER stress responses that promote tumor cell phagocytosis by professional antigen presenting cells (14). Moreover, Gholamin and colleagues have shown the promising therapeutic potential of targeting the CD47-SIRPα axis in patient-derived orthotopic xenograft models, where it reduces tumor growth in a variety of pediatric brain malignancies and inhibits metastasis (19). Accordingly, a number of immunological checkpoint inhibitors targeting the CD47-SIRPα axis are currently in clinical trials (58). So far, most efforts have been put on antibodies targeting CD47 or on Fc fusion proteins of the SIRPα ectodomain. However, a complication of effective targeting of the ubiquitously expressed CD47 with antibodies or fusion proteins containing an Fc is the occurrence of side effects such as anemia and thrombocytopenia. In this context, targeting of SIRPα, with its more confined expression pattern, may address some of these issues. And indeed, several anti-SIRPα antibodies are in active development in efforts to augment anti-tumor responses and overcome the significant off-target toxicities with anti-CD47 (56). Moreover, the nanobody format may bypass some of the safety concerns related to Fc-containing constructs. A direct therapeutic potential could be obtained if the nanobodies can modulate the CD47-SIRPα interaction, resulting in enhanced phagocytosis of cancer cells. The range of affinities detected for the identified nanobodies should in principle allow to interfere with the CD47-SIRPα interaction in a competitive manner, since the reported affinity for said interaction is in the sub-micromolar range (56).

In order to obtain sustained therapeutic effects, multivalency and lifetime extension of the nanobodies may be required, for example by genetically fusing the nanobody to a nanobody targeting serum albumin into a bispecific construct (59). Given the lower brain tumor uptake observed for bivalent nanobodies in this study, a sufficient level of accumulation in the tumor may be an attention point for multivalent and multispecific constructs. Of course, for therapeutic applications, the reduction in rapid tumor targeting for the bivalent constructs as detected here in the context of *in vivo* imaging, may be compensated for, by an increased accumulation in the tumor over time in case of life-time extended constructs (60). Of note, the size of multivalent and multispecific nanobody constructs is still smaller than full-sized antibodies, which may be beneficial for their brain targeting potential.

Next to counteracting the don’t-eat-me signal, nanobodies targeting SIRPα could also be used to deplete tumor-promoting myeloid cells, which facilitate GBM development and protect it from therapeutic treatments (61). Thereto, these nanobodies could be labeled with therapeutic radionuclides such as ^177^Lu, as we have shown before for nanobodies targeting mCD206 on tumor-associated macrophages (27). Alternatively, the nanobodies could be genetically coupled to an Fc-part that engenders antibody-dependent cellular cytotoxicity (ADCC), as we have documented for the depletion of tumor-infiltrating regulatory T cells (62). Of course, for such cell depletion approaches an important issue will be to avoid or at least minimize side-effects in peripheral organs where SIRPα is expressed, such as spleen, liver and lungs.

Overall, there is clear room for improvement and optimization of these nanobodies to increase their tumor targeting potential and protect the major antigen sinks (spleen, liver and lungs) to avoid possible side-toxicity effects. However, the notion that these nanobodies have reached their antigen in brain tumor-bearing mice is the first stepping stone towards further development. This is of particular importance given the high unmet medical need for brain pathologies at both diagnostic and therapeutic level.

## Data Availability Statement

All human and mouse scRNA-seq and CITE-seq datasets that we have used for this article can be accessed *via* our interactive webserver at www.brainimmuneatlas.org. All gene–cell count and cell annotation matrices can also be downloaded there. In addition, all mouse scRNA-seq and CITE-seq raw data, mouse gene–cell count matrices and human gene–cell count matrices have been deposited at GEO (NCBI) under accession number GSE163120. Raw sequencing reads of the human scRNA-seq and CITE-seq experiments have been deposited in the controlled access public repository European Genome-phenome Archive (EGA), under study accession number EGAS00001004871. Other data that support the findings of this study are available from the corresponding author upon request.

## Ethics Statement

The animal study was reviewed and approved by “Ethische Commissie Dierproeven” at Vrije Universiteit Brussel.

## Author Contributions

KV, ER, JP, and AP performed experiments. KV, ER, JP, AP, DK, ND, and IS have analysed data. GR, ND, KM, SM, and JG designed experiments. KV, ER, GR, and KM wrote the manuscript text. All authors have critically reviewed, read and agreed to the published version of the manuscript.

## Funding

This research project was realized with the support of Kom op tegen Kanker (project code ANI167) and a PhD scholarship grant from FWO to ER.

## Conflict of Interest

The authors declare that the research was conducted in the absence of any commercial or financial relationships that could be construed as a potential conflict of interest.

## Publisher’s Note

All claims expressed in this article are solely those of the authors and do not necessarily represent those of their affiliated organizations, or those of the publisher, the editors and the reviewers. Any product that may be evaluated in this article, or claim that may be made by its manufacturer, is not guaranteed or endorsed by the publisher.
