# Supplementary material for: Imaging of Glioblastoma Tumor-Associated Myeloid Cells Using Nanobodies Targeting Signal Regulatory Protein Alpha
Source: Front Immunol. 2021 Nov 30;12:777524. doi: 10.3389/fimmu.2021.777524 (PMC8669144; doi:10.3389/fimmu.2021.777524)
Supplement: Supplementary file 1 [file DataSheet_1.pdf]

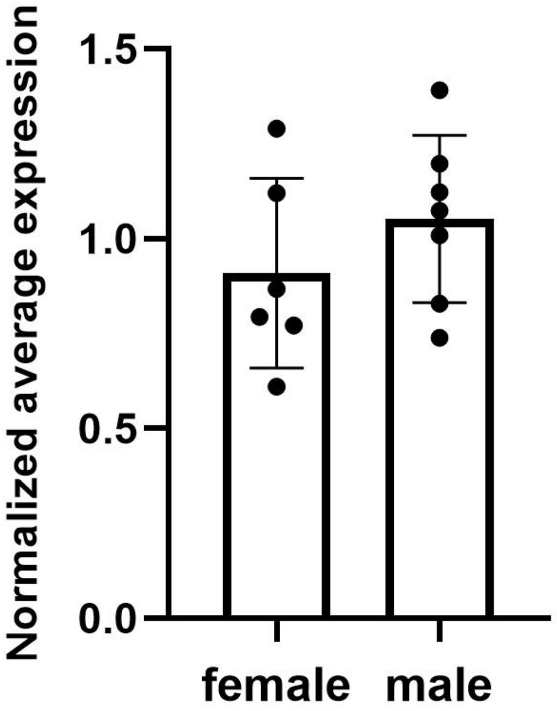

Supplementary Figure 1. SIRPα average gene expression in the GBM myeloid cell compartment (excl. mast cells) of 6 female and 7 male GBM patients, including patients with newly diagnosed (ND1-8) and recurrent (R1-5) GBM. No significant difference was observed between the two sexes. P-value=0.2964, evaluated by unpaired t-test.

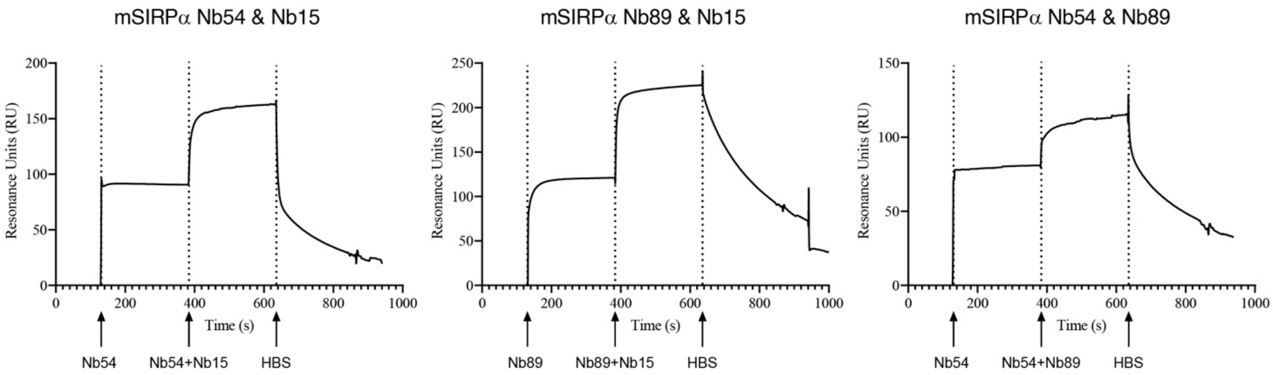

Supplementary Figure 2. Epitope binning to evaluate competition for the same epitope. Epitope binning by SPR: the sensorgrams of mSIRPα nanobodies binding to the recombinant antigen, upon sequential addition of two distinct nanobodies (as indicated by the arrows).

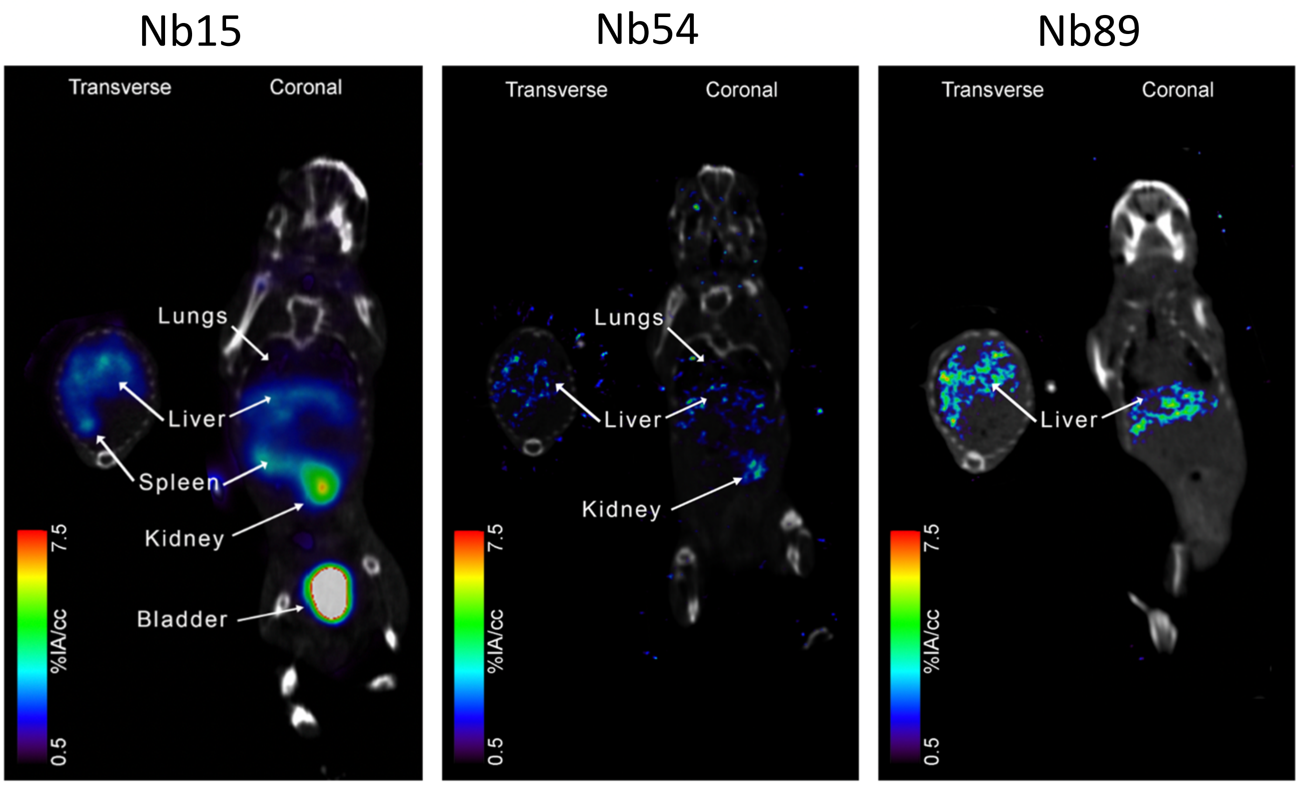

Supplementary Figure 3. Fused pinhole SPECT/micro-CT images of GL261 tumor-bearing mice injected with ^{99m}Tc-labeled anti- mSIRPα Nb clones 15, 54 and 89. Mice were imaged 1 hour post injection. Transverse and coronal views are shown, with slices chosen to reveal targeting to organs such as kidneys, lungs, spleen and liver. Slices that go through the brain tumor are shown in Figure 3. Results are representative of n=3 mice for each group.

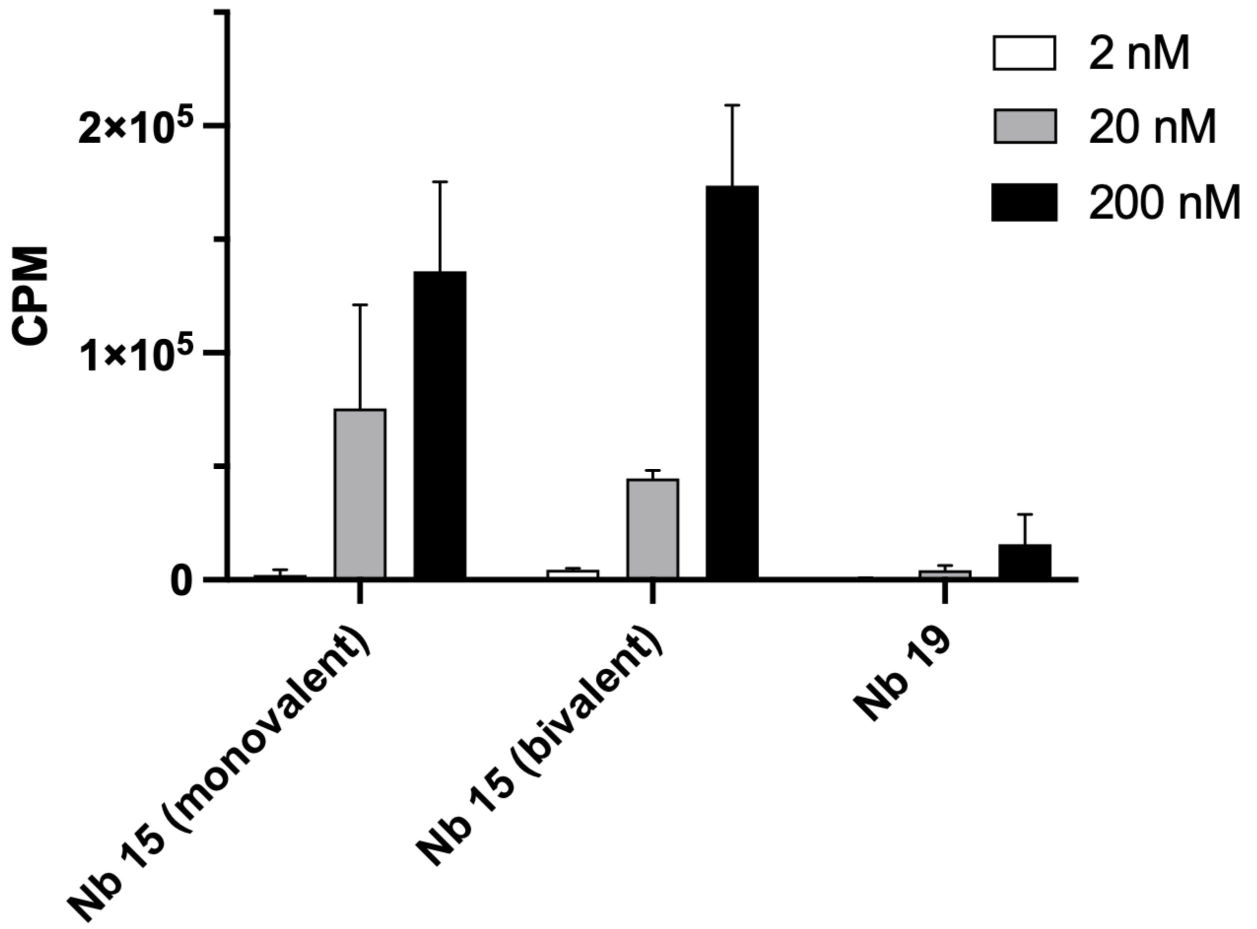

Supplementary Figure 4. Radioligand binding assay. mSIRPα-expressing cells were incubated with varying concentrations of radiolabeled Nb, with and without blocking by 100x molar excess of unlabeled Nb. Specific binding was calculated as $[CPM]_{Unblocked} - [CPM]_{Blocked}$.
CPM: counts per minute
