# Supplementary material for: Imaging of Glioblastoma Tumor-Associated Myeloid Cells Using Nanobodies Targeting Signal Regulatory Protein Alpha
Source: Front Immunol. 2021 Nov 30;12:777524. doi: 10.3389/fimmu.2021.777524 (PMC8669144; doi:10.3389/fimmu.2021.777524)
Supplement: Supplementary file 2 [file DataSheet_2.pdf]

Supplementary Table 1: Antibodies used in flow cytometry experiments: antigen, fluorophore, clone, lot number, ID (catalog number), manufacturer.

Antigen	Fluorophore	Clone	Lot number	ID	Manufacturer
CD11b	PE/cy7	M1/70	B227804	101216	BioLegend
CD172a (SIRPα)	PE	P84	B203321	144011	BioLegend
CD45	APC/cy7	30-F11	B242535	103115	BioLegend
CX3CR1	FITC	SA011F11	B272301	149022	BioLegend
F4/80	BV421	T45-2342	8330526	565411	BD Biosciences
HIS	PE	J095G46	B316502	362603	BioLegend
HA	AF488	16B12	1971419	A-21287	Thermo Fisher Scientific
I-A/I-E	PerCP/cy5.5	M5/114.15.2	B253463	107602	BioLegend
Ly-6B.2	AF647	7/4	0513R	MCA771A647	Biorad
